# Outcomes of isolated small bowel transplants in a single UK centre

**DOI:** 10.1186/2197-425X-3-S1-A357

**Published:** 2015-10-01

**Authors:** H Vollmer, E Harvey, G Barker, D Young

**Affiliations:** Oxford University Hospitals, Dept of Intensive Care, Oxford, United Kingdom

## Introduction

Small bowel transplantation is a curative procedure for the treatment of irreversible intestinal failure with the complications of parenteral nutrition or for extensive intra-abdominal disease requiring evisceration. 26 isolated small bowel transplants were performed in the UK in 2014 [1]. With advances in surgical techniques and the evolution of immunosuppressants, notably monoclonal antibody therapy, small bowel transplantation is increasingly considered feasible in many patients with intestinal failure.

## Objectives

To study the outcomes of small bowel transplants in our hospital and compare 90-day and 1-year mortality rates with the UK Transplant Registry Data and the US Scientific Registry of Transplant Recipients.

## Method

A review of all isolated small bowel transplants undertaken at the Churchill Hospital between October 2008 and July 2014. The medical notes and computer databases of the 28 patients were reviewed retrospectively and data extracted on patient demographics, indications for transplantation and outcomes. The primary outcomes were 90-day and 1-year mortality. Other outcomes recorded include length of ICU stay, length of hospital stay and ICU readmissions rates. A Kaplan Meier curve was constructed to analyse mortality.

## Results

The mean age was 41.5 ± 12.8 years and 57% were male. Inflammatory bowel disease (32.1%) and malignancy (21.4%) were the commonest indications for transplantation. Other indications were myopathies, radiation enteritis and ischaemic bowel. The 90-day mortality was 89.3% and 1-year mortality was 80.8%, with a mean survival time of 1413 ± 368 days. The mean post-operative length of stay on ICU was 7.2 ± 9.7 days. The mean overall ICU length of stay was 14.4 ± 16.5 days with a readmission rate of 44.8%. 85% of these patients were re-admitted more than once, from 10 to 1948 days following initial ICU discharge post-operatively.

## Conclusions

Our 90-day and 1-year mortality rates compare favourably with the UK Transplant Registry Outcomes [1], which has 90-day and 1-year mortality rates of 87% and 80% respectively and with the US Scientific Registry of Transplant Recipients Annual Data Report of 2013 [2]. The critical care input for these patients often extends beyond the initial post-operative period, as our data demonstrates. The 2012 SRTR report found that 86% of recipients were rehospitalised within 6 months of transplant [3]. Sepsis and rejection are common complications, which when accompanied by difficult vascular access can make the management of transplant patients challenging and resource intensive.

**Figure 1:**
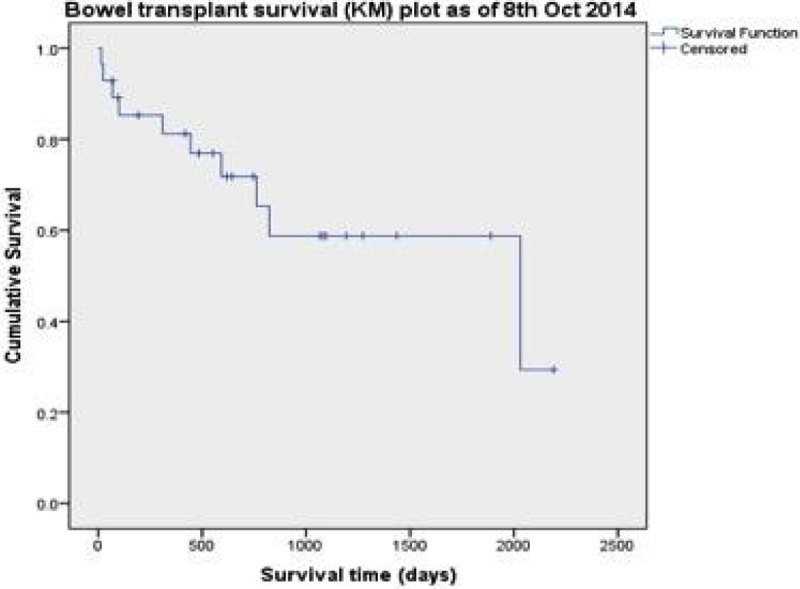
**Small Bowel Transplant Survival.**
